# Summary of the Standards, Options and Recommendations for the use of positron emission tomography with 2-[^18^F]fluoro-2-deoxy-D-glucose (FDP-PET scanning) in oncology (2002)

**DOI:** 10.1038/sj.bjc.6601088

**Published:** 2003-08-15

**Authors:** P Bourguet, M P Blanc-Vincent, A Boneu, L Bosquet, B Chauffert, C Corone, F Courbon, A Devillers, H Foehrenbach, J D Lumbroso, P Mazselin, F Montravers, J L Moretti, J N Talbot, and the FNCLCC PET-Scanning Committee

**Affiliations:** 1 Centre Eugène Marquis, Rennes, France; 2 FNCLCC, Paris, France; 3 Institut Claudius Regaud, Toulouse, France; 4 Centre Georges-François Leclerc, Dijon, France; 5 Centre René Huguenin, Saint-Cloud, France; 6 Centre Eugène Marquis, Rennes, France; 7 Hôpital d'Instruction des Armées du Val de Grâce, Paris, France; 8 Institut Gustave Roussy, Villejuif, France; 9 Hôpital d'Instruction des Armées Sainte Anne, Toulouse, France; 10 Hôpital Tenon, Paris, France; 11 Hôpital Avicenne, Bobigny, France

**Keywords:** positron emission tomography, practice guideline

When positron emission tomography (PET) scanning was introduced at the end of the 1970s, its technical characteristics and biological potential aroused immediate interest. The available tracers at time (isotopes of oxygen, nitrogen, and carbon) made it possible to study blood flow, regional oxygen consumption, the main metabolic pathways and ligand–receptor interactions in the brain, heart and other major organs, without physiological perturbations. Although the promise of the technique was fulfilled, its use has not developed as rapidly as expected.

Positron emission tomography scanning was initially used to study the brain and the heart, but today it is used mainly in oncology. This is partly due to technological developments that allow whole-body examinations. There is also a growing number of publications suggesting that this technique is useful in the management of many cancers, from initial staging to post-therapeutic follow-up.

The tracer generally used is 2-[^18^F]fluoro-2-deoxy-D-glucose (FDG), which is a glucose analogue that competes with glucose at the level of transmembrane transporters. Although other tracers have been proposed ([^11^C]methionine, [^11^C]tyrosine and [^11^C]thymidine), their use has not yet been validated, and the carbon-11 label is a limiting factor for extensive routine use. Nearly 70 years ago, Warburg demonstrated an increase in glycolytic activity in cancer cells, and this is the basis for use of FDG in oncology. Briefly, in most cancers, neoplastic transformation induces an increase in glucose transporters (particularly GLUT1) and in the activity of glycolytic enzymes (particularly hexokinase). These changes are responsible for an increase in glycolytic activity in cancer cells, under both aerobic and anaerobic conditions. The glycolytic activity is related to the viable tumour cell mass, as the increase in glucose transport reflects cell proliferation. Accumulation of glucose is not specific to malignant tumours but can also be increased in benign tumours and in inflammatory diseases, such as sarcoidosis and granulomatosis.

In 2001, there were only four operational PET scanners in France, dedicated to clinical use. Since then, the Government has authorised the installation of about 40 sites, with a final objective of 60 PET scanners so as to provide adequate access throughout the country.

The most important question about the use of PET scanning in oncology is: ‘What is its usefulness in comparison with other imaging techniques?’ The answer requires not only comparing the performance of PET scanning with that of other imaging techniques, but also evaluating the impact of use of PET on the management of patients with cancer. Although, many studies are under way, only a few publications specifically addressing the question are available.

As with most medical imaging techniques, the clinical use of PET has developed before its efficacy and efficiency have been clearly demonstrated. The fields of application of PET scanning are evolving continuously with new research findings. However, the rapid pace of technological improvements to PET scanning results in an ever increasing list of applications, but this also prevents the accumulation of convincing data for evaluation. In this context, it was decided that clinical practice guidelines were needed to define the potential and recognised indications for FDG-PET scanning in oncology.

## OBJECTIVES

The objective was to review the available scientific data and to develop the Standards, Options, and Recommendations (SORs) for the role of and indications for FDG-PET scanning in oncology. The main steps in patient care that were studied were diagnosis of the primary disease, initial and secondary metastatic assessment, evaluation of treatment response, and detection of recurrent disease. The recommendations made relate to the primary cancer sites defined as priorities on the basis of the available scientific data: cancers of lung and pleural, melanoma, gynaecological cancers, gastrointestinal cancers, head-and-neck cancers, urological cancers, lymphomas, soft-tissue and bone sarcomas, and cancers of the thyroid, and also carcinomas of unknown primary site. For some cancer sites, the working group considered that an evaluation was either not timely or that the available data were inadequate. These topics, in particular cerebral tumours and childhood cancers, will be addressed when these recommendations are updated.

## METHODS

The details of the methodology have been published previously (Fervers *et al*, 2001). For this particular SOR, a multidisciplinary group of experts was set up by the French National Federation of Cancer Centres (FNCLCC) and the French Society for Biophysics and Nuclear Medicine (Société Française de Biophysique et Médecine Nuclear, SFBMN) to critically appraise the available evidence on the role of and indications for FDG-PET scanning in oncology.

Literature searches were performed for each cancer site in Medline®, from January 1996 to November 2001, and in the Cochrane® Library, Issue 3, 1999. The Cancerlit® database and the proceedings from American Society of Clinical Oncology conferences were also searched. The search excluded articles in languages other than English and French, as well as *in vitro* and animal studies. Studies in which tracers other than FDG were used were not specifically sought, although studies comparing FDG with other tracers were included for certain cancer sites, when they provided data for the relevant outcomes. The review met with a recurrent difficulty: multiple publication in different journals of the same study, with an increasing number of patients, and sometimes with the authors in a different order. In this situation, only the last publication, including the largest number of patients, was retained for this report.

The literature search was complemented with personal references supplied by the experts. In certain chapters, references published after November 2001 were added when the working group considered it necessary, especially when the new references had an impact on the definition of a standard or an option. The data analysis also included three reports of evaluations and recommendations for FDG-PET scanning (Adams *et al*, 1999; Robert and Milne, 1999; AETMIS, Agence d'évaluation des technologies et des modes d'intervention en santé, 2001)Please check change in year from 2000 to 2001 OK and the report of a German consensus conference (Reske and Kotzerke, 2001).

The working group selected and critically appraised pertinent references and then proposed the ‘Standards’, ‘Options’, and ‘Recommendations’ for the role of and indications for FDG-PET scanning in oncology, based on either the best available evidence or expert agreement.

‘Standards’ identify clinical situations for which there exist strong indications or contraindications for a particular FDG-PET application and ‘Options’ identify situations for which there are several alternatives, none of which have shown clear superiority over the others (Table 1

**Table 1:** Definition of Standards, Options and Recommendations

Standards	Procedures or treatments that are considered to be of benefit, inappropriate or harmful by unanimous decision based on the best available evidence
	
Options	Procedures or treatments that are considered to be of benefit, inappropriate or harmful by a majority, based on the best available evidence.
	
Recommendations	Additional information to enable the available options to be ranked using explicit criteria (e.g. survival, toxicity) with an indication of the level of evidence

). In any SOR, there can be several ‘Options’ for a given clinical situation. ‘Recommendations’ enable the ‘Options’ to be weighted according to the available evidence. Several FDG-PET applications can be recommended for the same clinical situation, so that clinicians can make a choice according to specific clinical parameters, for example, local circumstances, skills, equipment, resources, and patient preferences. Adapting the SORs to a local situation is possible if the reason for the choice is sufficiently transparent and this is crucial for successful implementation. Inclusion of patients in clinical trials is an appropriate form of patient management in oncology and is recommended frequently within the SORs, particularly in situations where evidence is too weak to support a particular FDG-PET application.

The type of evidence underlying any ‘Standard’, ‘Option’, or ‘Recommendation’ is indicated using a classification developed by the FNCLCC based on previously published models. The level of evidence depends not only on the type and quality of the studies reviewed, but also on the concordance of the results (Table 2

**Table 2:** Definition of level of evidence

*Level A*
There exist a high-standard meta-analysis or several high-standard randomised clinical trial which give consistent results

*Level B*
There exist clinical trials (therapeutic trials, quasiexperimental trials or comparisons of populations) for which the results are consistent when considered together

*Level C*
There exist clinical trails (therapeutic trials, quasiexperimental trails, or comparisons of populations) for which the results are not consistent when considered together

*Level D*
Either the scientific data do not exist or there is only a series of cases

*Expert agreement*
The data do not exist for the method concerned, but the experts are unanimous in their judgement

). When no clear scientific evidence exists, judgment is made according to professional experience and consensus of the working group (‘expert agreement’).

In this particular situation, that is, a diagnostic test, it is sometimes difficult to classify levels of evidence. In addition, PET scanning is an emerging technique, for which many indications are still being evaluated. The working group, therefore, decided to identify not only standards and options for protocols being evaluated but also indications that require confirmation. The standards are based on levels of evidence A or B and represent indications for which the working group considered that PET scanning is essential for the care of patients. The options are usually based on a high level of evidence (B2), whereas the indications that require confirmation are those for which published data are scarce or insufficient (levels of evidence C, D, and expert agreement). For certain indications, despite a low level of evidence, the clinical usefulness of PET scanning was considered by the working group to be high, thus the indication is classified as an option (expert agreement).

The document containing the SORs was then reviewed by a group of independent experts (see the Appendix) and after taking into consideration their comments, the guidelines were validated by the working group.

This English-language version is based on the summary version, which was itself based on the French full text version (Bourguet *et al*, 2003). The French full text and summary versions are available on the FNCLCC web site (http://www.fnclcc.fr/sci/sor/bonnes_ pratiques/tep_fdg.htm).

A working group has been set up to monitor new scientific data on FDG-PET systematically. These clinical practice guidelines will be updated when new evidence becomes available or if there is a new consensus among the experts. In addition, patient-targeted information is being developed by the SOR SAVOIR PATIENT project, based on the specialist information (available late 2003). The SORs for use of FDG-PET scanning in oncology are summarised in Table 3

**Table 3:** Summary of Standards, Options, and Recommendations for FDP-PET scanning

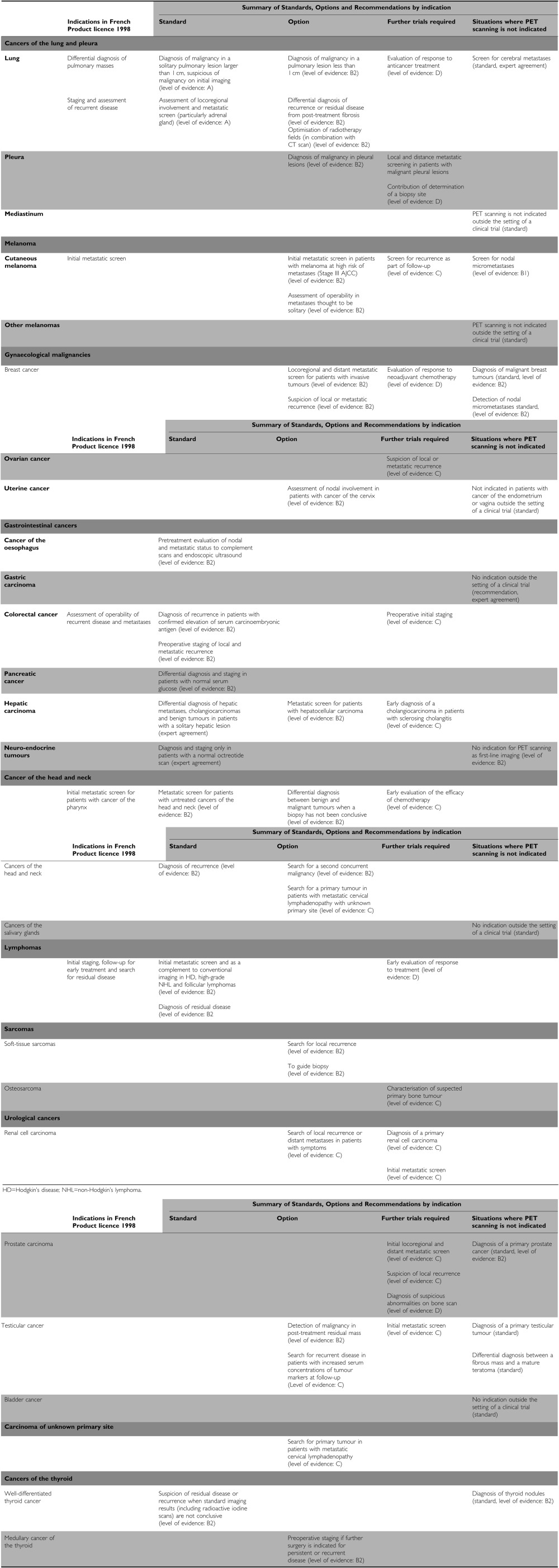

.

## Appendix

### Reviewers

C Allavena (Centre Sainte-Catherine de Sienne, Nantes, France), C Aractingi (Hôpital Tenon, Paris, France), R Arriagada (IRAM, Santiago, Chile), JM Bachaud (Institut Claudius Regaud, Toulouse, France), JP Basuyau (Centre Henri Becquerel, Rouen, France), V Beckendorf (Centre Alexis Vautrin, Vandœuvre-lès-Nancy, Nancy, France), A Bertrand (CHU Brabois, Vandœuvre-lès-Nancy, Nancy, France), JY Blay (Centre Léon Bérard, Lyon, France), A Botton (Clinique Sainte Marie, Pontoise, France), A Brémond (Centre Léon Bérard, Lyon, France), P Brunotte (Centre Georges-François Leclerc, Dijon, France), B Buecher (CHU Nantes, Nantes, France), MF Carette (Hôpital Tenon, Paris, France), F Chomy (Institut Bergonié, Bordeaux, France), C Chouaid (Hôpital Saint Antoine, Paris, France), B Coiffier (Centre Hospitalier Lyon Sud, Pierre-Bénite, France), P Colombat (CHU Hôpital Bretonneau, Tours, France), T Conroy (Centre Alexis Vautrin, Vandœuvre-lès-Nancy, Nancy, France), M Dahan (CHU Purpan, Toulouse, France), O De Dreuille (Hôpital Val de Grâce, Paris, France), D Delbeke (Vanderbilt University Medical Center, Nashville, Tennessee, USA), J Delgado (Centre d'Imagerie Radio-isotopique, La Rochelle, France), F Demard (Centre Antoine Lacassagne, Nice, France), MH Dilhuydy (Institut Bergonié, Bordeaux, France), B Dupas (CHU Hôtel-Dieu, Nantes, France), N Eche (Institut Claudius Regaud, Toulouse, France), V Edeline (Institut Curie, Paris, France), D Elias (Institut Gustave Roussy, Villejuif, France), S Evrard (Institut Bergonié, Bordeaux, France), PL Etienne (Clinique Armoricaine, Saint Brieuc, France), E François (Centre Antoine Lacassagne, Nice, France), G Frija (Société Française de Radiologie et Imagerie Médicales, Paris, France), G Ganem (Centre Jean Bernard, Le Mans, France), JP Gérard (Centre Antoine Lacassagne, Nice, France), F Giammarile (Centre Léon Bérard, Lyon, France), J Giron (CHU Purpan, Toulouse, France), G Goerres (Klink und Poliklinik für Nuklearmedizein Universitäts Spital, Zürich, Switzerland), S Goldman (Hôpital Erasme, Brussels, Belgium), P Grenier (Hôpital Pitié Salpetrière, Paris, France), JJ Grob, (Hôpital La Timone, Marseille, France), B Guilloneau (Institut Mutualiste Montsouris, Paris, France), M Janier (Hôpital Edouard Herriot, Lyon, France), D Jeanbourquin (Hôpital Percy, Clamart), G Jérusalem (CHU, Liege, Belgium), B Lambert (Clinique Bordeaux Nord, Bordeaux, France), P Lasser (Institut Gustave Roussy, Villejuif, France), H Lauche (Clinique de Clémentville, Montpellier, France), R Lavayssière (Centre d'Imagerie Paris-Nord, Sarcelles, France), F Lazorthes (CHU, Toulouse, France), P Lederlin (CHU Brabois, Vandœuvre-lès-Nancy, Nancy, France), T Leludec (Hospices Civils de Lyon, Lyon, France), H Lena (CHU, Rennes, France), A Lortholary (Centre Paul Papin, Angers, France), JP Lotz (Hôpital Tenon, Paris, France), F Marchal (Centre Alexis Vautrin, Vandœuvre-lès-Nancy, Nancy, France), X Marchandise (CHU, Lille, France), P Martin (Centre Bourgogne, Lille, France), Y Martinet (CHU Brabois, Vandœuvre-lès-Nancy, Nancy, France), M Marty (Institut Gustave Roussy, Villejuif, France), J Maublant (Centre Jean Perrin, Clermont-Ferrand, France), Y Merrouche (CHU, Saint-Etienne, France), JP Muratet (Centre Jean Bernard, Le Mans, France), H Orfeuvre (Centre Hospitalier Fleyriat, Bourg en Bresse, France), P Paulus, (CHR La Citadelle, Liege, Belgium), S Petit (Centre Bourgogne, Lille, France), M Perol (Hôpital de la Croix Rousse, Lyon, France), T Philip (Centre Léon Bérard, Lyon, France), MF Pichon (Centre René Huguenin, Saint-Cloud, France), JD Rain (Hôpital Saint Louis, Paris, France), JC Rambaud (Hôpital Lariboisière, Paris, France), JL Raoul (Centre Eugène Marquis, Rennes, France), R Regal (Clinique de Clémentville, Montpellier, France), B Roullet (Centre Eugène Marquis, Rennes, France), N Rouverand (Clinique de la Louvière, Lille, France), H Rubie (Hôpital Purpan, Toulouse, France), M Schlumberger (Institut Gustave Roussy, Villejuif, France), P Solal-Celigny (Centre d'Oncothérapie et Hématologie, Le Mans, France), D Spaeth (Centre Alexis Vautrin, Vandœuvre-lès-Nancy, Nancy, France), J Stines (Centre Alexis Vautrin, Vandœuvre-lès-Nancy, Nancy, France), G Storme, OECI (Brussels, Belgium), E Tiret, (Hôpital Saint Antoine, Paris, France), A Villers (Hôpital Huriez, Lille, France), and JJ Voigt (Institut Claudius Regaud, Toulouse, France).
